# Comparison of Allergic Rhinitis Treatments on Utilities and Quality of Life: A MASK‐air Study

**DOI:** 10.1111/cea.70335

**Published:** 2026-05-06

**Authors:** Nuno Lourenço‐Silva, Bernardo Sousa‐Pinto, Antonio Bognanni, Ana Margarida Pereira, João Coutinho‐Almeida, Rita Amaral, Luisa Brussino, Mattia Giovannini, Bilun Gemicioglu, Violeta Kvedariene, Desiree E. Larenas‐Linnemann, Manuel Marques‐Cruz, Nikolaos G. Papadopoulos, Nhân Pham‐Thi, Frederico S. Regateiro, Sanna K. Toppila‐Salmi, Boleslaw Samolinski, Joaquin Sastre, Luís Taborda‐Barata, Arunas Valiulis, Leticia de las Vecillas, Maria Teresa Ventura, Oliver Pfaar, João A. Fonseca, Torsten Zuberbier, Ludger Klimek, Jean Bousquet, Rafael José Vieira

**Affiliations:** ^1^ MEDCIDS – Department of Community Medicine, Information and Health Decision Sciences, Faculty of Medicine University of Porto Porto Portugal; ^2^ CINTESIS@RISE – Health Research Network, Faculty of Medicine University of Porto Porto Portugal; ^3^ Clinical Epidemiology and Research Center (CERC) Humanitas University and Humanitas Research Hospital Milan Italy; ^4^ Allergy Unit Instituto and Hospital CUF Porto Portugal; ^5^ Department of Cardiovascular and Respiratory Sciences, Porto Health School Polytechnic Institute of Porto Porto Portugal; ^6^ Department of Women's and Children's Health, Paediatric Research Uppsala University Uppsala Sweden; ^7^ Department of Medical Sciences University of Torino Torino Italy; ^8^ Allergy and Clinical Immunology Unit Mauriziano Hospital Torino Italy; ^9^ Allergy Unit Meyer Children's Hospital IRCCS Florence Italy; ^10^ Department of Health Sciences University of Florence Florence Italy; ^11^ Department of Pulmonary Diseases, Cerrahpaşa Faculty of Medicine Istanbul University‐Cerrahpaşa Istanbul Turkey; ^12^ Department of Pulmonary Diseases, Institute of Pulmonology and Tuberculosis Istanbul University‐Cerrahpaşa Istanbul Turkey; ^13^ Clinic of Chest Diseases and Allergology, Faculty of Medicine, Institute of Clinical Medicine Vilnius University Vilnius Lithuania; ^14^ Department of Pathology, Faculty of Medicine, Institute of Biomedical Sciences Vilnius University Vilnius Lithuania; ^15^ Center of Excellence in Asthma and Allergy Médica Sur Clinical Foundation and Hospital México City Mexico; ^16^ Allergy Department, 2nd Pediatric Clinic University of Athens Athens Greece; ^17^ Ecole Polytechnique de Palaiseau Palaiseau France; ^18^ IRBA (Institut de Recherche Bio‐Médicale des Armées) Bretigny sur Orge France; ^19^ Université Paris Cité Paris France; ^20^ Center for Allergy and Clinical Immunology, Alésia Paris France; ^21^ Allergy and Clinical Immunology Department, Hospitais da Universidade de Coimbra Unidade Local de Saúde de Coimbra Coimbra Portugal; ^22^ Center for Innovative Biomedicine and Biotechnology (CIBB), Faculty of Medicine University of Coimbra Coimbra Portugal; ^23^ Faculty of Medicine, Institute of Immunology University of Coimbra Coimbra Portugal; ^24^ UBIAir – Clinical & Experimental Lung Centre and CICS‐UBI Health Sciences Research Centre University of Beira Interior Covilhã Portugal; ^25^ Department of Otorhinolaryngology University of Eastern Finland and the North Savo Wellbeing Services County Kuopio Finland; ^26^ Department of Allergy, Skin and Allergy Hospital, Inflammation Center Helsinki University Hospital and University of Helsinki Helsinki Finland; ^27^ Department of Prevention of Environmental Hazards, Allergology and Immunology Medical University of Warsaw Warsaw Poland; ^28^ Allergy Service, Fundacion Jimenez Diaz Universidad Autonoma de Madrid, CIBERES‐ISCIII Madrid Spain; ^29^ RISE‐Health, Faculty of Health Sciences, and UBIAir‐Clinical & Experimental Lung Centre University of Beira Interior Covilhã Portugal; ^30^ Department of Immunoallergology Unidade Local de Saúde, Cova da Beira Covilhã Portugal; ^31^ Clinic of Asthma, Allergy and Chronic Lung Diseases Vilnius Lithuania; ^32^ Institute of Clinical Medicine and Institute of Health Sciences Medical Faculty of Vilnius University Vilnius Lithuania; ^33^ Department of Allergy Hospital La Paz Institute for Health Research (IdiPAZ) Madrid Spain; ^34^ University of Bari Medical School Bari Italy; ^35^ Institute of Sciences of Food Production National Research Council (ISPA‐CNR) Bari Italy; ^36^ Section of Rhinology and Allergy, Department of Otorhinolaryngology, Head and Neck Surgery University Hospital Marburg, Philipps‐Universität Marburg Marburg Germany; ^37^ Institute of Allergology, Charité – Universitätsmedizin Berlin Corporate Member of Freie Universität Berlin and Humboldt‐Universität zu Berlin Berlin Germany; ^38^ Fraunhofer Institute for Translational Medicine and Pharmacology ITMP, Immunology and Allergology Berlin Germany; ^39^ Department of Otolaryngology, Head and Neck Surgery Universitätsmedizin Mainz Mainz Germany; ^40^ Center for Rhinology and Allergology Wiesbaden Germany; ^41^ ARIA (Allergic Rhinitis and its Impact on Asthma) Montpellier France; ^42^ MASK‐air Montpellier France

## Abstract

**Background:**

Allergic rhinitis displays a relevant impact on quality of life. Medications used in the treatment of rhinitis have been assessed on their impact on rhinoconjunctivitis‐related quality of life, but not on generic health‐related quality of life metrics, such as utilities or EQ‐5D visual analogue scale (VAS) levels. This study aimed to compare different medication classes and individual medications on utilities and EQ‐5D VAS levels using data from a mobile app.

**Methods:**

We conducted an observational study using direct patient data from the MASK‐air mobile application, collected between May 2015 and December 2024. We compared rhinitis medication classes and individual medications on health utilities (computed from the EQ‐5D‐5L questionnaire) and the EQ‐5D VAS. To account for confounding, we employed inverse probability treatment weighting based on propensity scores, adjusting for demographics, baseline symptom control, and asthma status.

**Results:**

The study analysed 69,973 observations with EQ‐5D VAS data and 842 observations with utility data. At the medication class level, fixed combinations of intranasal antihistamines and corticosteroids were associated with improvements in EQ‐5D VAS (mean difference = 1.900; 95% CI = 1.316–2.484) and utilities (mean difference = 0.022; 95% CI = −0.015 to 0.059) compared with oral antihistamines (OAH). Intranasal antihistamines were associated with lower EQ‐5D VAS and utility scores than other intranasal treatments. For individual medications, mometasone was associated with a lower EQ‐5D VAS than budesonide and fluticasone furoate, while fexofenadine and levocetirizine tended to be associated with lower VAS values than other OAH.

**Conclusion:**

Fixed combinations of intranasal antihistamines and corticosteroids were associated with better quality‐of‐life than oral antihistamines and intranasal antihistamines. These findings could support future cost‐effectiveness analyses.

## Key Points

- Fixed INAH+INCS combinations improved EQ‐5D VAS and utilities versus oral antihistamines.
- Intranasal antihistamine monotherapy showed the lowest quality‐of‐life scores across medication classes.
- Individual drug differences were small; mometasone, fexofenadine and levocetirizine had lower VAS.

## Graphical abstract

This mHealth study has shown that fixed combinations of intranasal antihistamines and corticosteroids were associated with higher quality of life than oral antihistamines and intranasal antihistamines. Both intranasal corticosteroids and oral antihistamines were associated with higher quality of life than intranasal antihistamines.

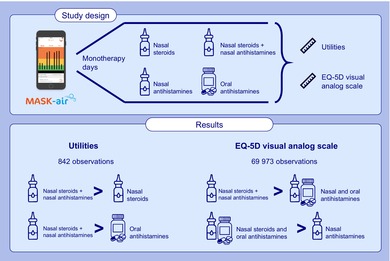

## 1 Introduction

Allergic rhinitis (AR) is one of the most prevalent chronic conditions worldwide, with substantial impact on quality of life, daily functioning, sleep and work productivity [1, 2, 3]. This condition also imposes an important socioeconomic burden through healthcare utilisation and indirect costs [2].

Several pharmacological treatments for AR are available, including oral antihistamines (OAH), intranasal corticosteroids (INCS), intranasal antihistamines (INAH), and fixed combinations of INAH and INCS (INAH+INCS) [1, 3, 4, 5, 6, 7]. Randomised controlled trials (RCTs) have vastly demonstrated medication efficacy in the symptom control of AR [8, 9, 10]. One of the outcomes most frequently explored by RCTs is the impact on quality of life. These trials have examined quality‐of‐life but such an evaluation has mostly focused on rhinoconjunctivitis‐related quality of life, and has not considered generic health‐related quality‐of‐life measures. The latter are important as they can allow the computation of utilities for the corresponding health states [9, 11]. The measurement of utilities—quantitative measures of the population's preferences for the corresponding health states—is particularly relevant for conducting cost‐utility studies, comparing medications not only in terms of costs but also in terms of effectiveness. Quantifying utilities also allows for comparing different diseases in terms of their impact on quality of life. On the other hand, the impact of AR medications on quality of life in a context different from that of RCTs (e.g., less severe participants) has been insufficiently explored. Mobile health (mHealth) technologies provide new opportunities to collect ‘real‐world’ data directly from patients. The MASK‐air mobile application has been widely used in AR research, and its patient‐reported outcome measures may help compare the impact of different medications on quality of life, overcoming some of the limitations of current RCTs [12, 13].

Therefore, the present study aimed to compare different AR medications on their impact on quality of life, as measured by utilities and the EQ‐5D visual analogue scale (VAS). Results of this study will inform the recommendations of the Allergic Rhinitis and its Impact on Asthma (ARIA) ‐ European Academy of Allergy and Clinical Immunology (EAACI) 2024–2025 guidelines, as it provides utility data to compute quality‐adjusted life years and assess the cost‐effectiveness of the interventions.

## 2 Methods

### 2.1 Study Design

We performed a retrospective observational study using direct patient data from the MASK‐air mobile app. For several AR medication classes and individual monotherapy medications, we compared the underlying utility values and EQ‐5D VAS values.

### 2.2 Setting and Participants

The MASK‐air app, accessible at www.mask‐air.com, is a free digital health tool available in over 30 countries and 20 languages [4, 6, 14]. MASK‐air patients have either been enrolled by physicians or have started using the app of their own accord. We included MASK‐air users (i) aged between 16 and 75 years (or more than 13 years in countries where the age of digital consent is lower) and (ii) who had self‐reported AR. Data were collected from May 15, 2015 to December 31, 2024.

We assessed MASK‐air data (days) (i) in which users had answered the EQ‐5D VAS, and (ii) in which there was reported use of INAH, INCS, INAH + INCS or OAH in monotherapy. In fact, we decided not to include days of co‐medication (i) after a preliminary analysis which indicated that monotherapy and co‐medication days are different in their patterns of patient‐reported outcome measures (PROMs; Table S1), and (ii) considering the need for more advanced approaches to disentangle the different patterns of co‐medication use and their relationship with patients' symptoms (co‐medication patterns represent distinct therapeutic strategies that require separate analytical approaches). Of note, to reflect patients' perspectives, we defined ‘co‐medication’ as strategies that imply the use of more than one formulation. Therefore, fixed combinations of INAH+INCS (e.g., azelastine‐fluticasone and olopatadine‐mometasone) were not considered to be part of co‐medication as they are used as a single formulation [15].

### 2.3 Variables

MASK‐air includes a daily monitoring questionnaire in which patients report their symptoms, medication use and EQ‐5D VAS. Symptom VASs range from 0 to 100, with higher values indicating more severe symptoms (Table S2). Medication is reported through a customised scroll list that displays all available prescribed and over‐the‐counter medications for each country where MASK‐air is available. In addition to the daily monitoring questionnaire, users can answer additional questionnaires, including the EQ‐5D‐5L (however, in contrast to the EQ‐5D VAS, users are not requested to complete the full EQ‐5D‐5L questionnaire on a daily basis). EQ‐5D‐5L is a standardised measure of health‐related quality of life developed by the EuroQol Group and is the most widely used multi‐attribute utility instrument [16]. It assesses five domains and produces a five‐digit health state profile that can be converted into a single index value (utilities), quantifying the population's preferences for the respective health states. The EQ‐5D‐5L questionnaire also includes a visual analogue scale (VAS) to enable patients to self‐assess their health status [17].

Comparisons were performed at two levels: (i) medication classes (OAH, INCS, INAH and fixed combinations INAH + INCS), and (ii) individual medications: among oral and nasal medications, we performed comparisons between the most frequently reported individual medications (e.g., loratadine vs. rupatadine). We considered only days on which the aforementioned medications were used in monotherapy. In line with patients' perspectives, we defined ‘monotherapy days’ as those when only one AR drug formulation was reported. In this context, even though they contain two active compounds, fixed combinations of INAH + INCS were considered as monotherapy (patients use them in a single formulation).

The assessed outcomes were health utilities, computed using the EQ‐5D‐5L instrument [17], and the EQ‐5D VAS. These are two complementary measures. The EQ‐5D‐5L utility index has been derived from the five EQ‐5D‐5L dimensions (mobility, self‐care, usual activities, pain/discomfort and anxiety/depression), each recorded on a five‐level scale on the respective day and converted into a single preference‐based value using population‐specific value sets for each assessed country. Utility values are anchored at 1.0 for full health and 0.0 for a state equivalent to death, with values below 0 representing states considered worse than death. On the other hand, the EQ‐5D VAS is a pragmatic and sensitive PROM that allows patients to rate their overall health status on a 0–100 scale, where 0 represents the ‘worst imaginable health state’ and 100 the ‘best imaginable health state’ [18]. A previous MASK‐air study has estimated a minimal important difference of 10 points for EQ‐5D VAS [19].

### 2.4 Ethics

MASK‐air is both a non‐CE‐marked and a Class IIa device under the Medical Device Regulation, and adheres to the General Data Protection Regulation (GDPR) [4, 20]. Data are provided anonymously by users, and geolocation information is protected using k‐anonymity to ensure privacy [21]. Independent ethics committee approval was not required for the present study because (i) the use of MASK‐air data for research had previously been authorised by an independent review board (Köln‐Bonn, Germany; reference number 17‐069) [22], (ii) all data were anonymised before analysis, and (iii) users had provided informed consent for the use of their data in research through acceptance of the terms of use.

### 2.5 Statistical Analysis

Categorical variables were described using absolute and relative frequencies, while continuous variables were described using medians and interquartile ranges or means and standard deviations. When responding to the MASK‐air daily monitoring questionnaire, it is not possible to skip any of the questions, and data are saved to the dataset only after the final answer. This precludes any missing data within each questionnaire. All analyses were conducted in R version 4.5.1, using the tidyverse and lme4 packages for data management, propensity score estimation, and prediction.

### 2.6 Propensity Score Estimation and Weighting

We compared different medication classes and individual medications on their utility and EQ‐5D VAS values. To reduce confounding, we estimated propensity scores (PS) for each treatment using mixed‐effects logistic regression models. These models included several variables as fixed effects: gender, asthma status and baseline disease impact. We also included the Combined Symptom‐Medication Score (CSMS) of the day before treatment and added a variable to account for patterns of data registration within the app, classifying observations into five distinct categories: (i) first day of using the app, (ii) second consecutive day of using the app, (iii) other consecutive days, (iv) first non‐consecutive data point, and (v) subsequent non‐consecutive datapoints [23]. The identification of the patient and the month of the year (to account for seasonality) were set as random effects, so that observations were clustered by patient and month. Covariates were chosen based on clinical relevance and previous literature on AR treatment allocation and outcomes. In particular, the CSMS of the previous day has been used in previous studies as a proxy for the AR control level before medication use [15]. In fact, the CSMS evaluates the daily control of AR when accounting for the reported symptoms and medication use, according to the following equation [24]:

$$\begin{array}{c} {}[\left(0.037\times \mathrm{VAS}\;\text{Global Symptoms}\right)+\left(0.033\times \mathrm{VAS}\;\text{Eyes}\right)\\ +\left(0.020\times \mathrm{VAS}\;\text{Nose}\right)+\left(0.027\times \mathrm{VAS}\;\text{Asthma}\right)\\ +\left(0.450\;\text{if Azelastine}-\text{Fluticasone is used}\right)\\ +\left(0.424\;\text{if nasal steroids}\;\mathrm{are}\;\text{used}\right)\\ +\left(0.243\;\text{if asthma medication is used}\right)\\ +\left(0.380\;\text{if other rhinitis relief medication is used}\right)]\times 7.577 \end{array}$$

On the other hand, the baseline impact corresponds to the patient's ARIA score (range of 0–4; assesses the number of different ways in which allergy symptoms affect users) and evaluates the severity of the disease [13].

We then applied inverse probability of treatment weighting [25, 26]. Stabilised weights were calculated by dividing the marginal probability of treatment by the conditional (predicted) probability of treatment. This approach ensures unbiased estimation of treatment effects under the assumption of no unmeasured confounding, positivity, and correct model specification.

### 2.7 Outcome Models

Mixed‐effects linear regression models were fitted to estimate mean differences in both EQ‐5D VAS and utilities across treatments. Asthma status was added as a covariate, and patient identification and month were added as random effects. Robust standard errors were computed to account for the variability introduced by the weighting procedure. Results are reported as adjusted mean differences with 95% confidence intervals (CI).

Importantly, we have built models only for comparisons with at least 50 observations. Whenever model convergence was not reached, we attempted an alternative approach: building similar models without the random effects component (i.e., non‐mixed methods regression models). In that approach, to avoid multiple observations from the same user, we randomly selected one observation per patient per day.

## 3 Results

### 3.1 Characteristics of the Assessed Sample

The study analysed (i) 69,973 daily observations with valid EQ‐5D VAS responses from 4276 distinct users, and (ii) 842 observations with utility scores from 416 users (Table 1; Figure S1). In the EQ‐5D VAS sample, the median age was 39 years, and a majority of users were female (56.2%). Asthma was a common comorbidity, reported by 30.8% of users. In contrast, in the utility sample, the median age was 37 years, and 60.8% were female. In this group, 35.6% of users reported asthma. Country distribution of users and analysed days is available in Table S3. As mentioned, assessed days involve the use of monotherapy, partly because co‐medication days were associated with different PROM patterns (more severe symptoms and lower satisfaction) (Table S1).

**TABLE 1:** Characteristics of the assessed participants and their reported days.

Variable	Assessed sample	
	EQ‐5D VAS (*N* users = 4276)	Utility (*N* users = 416)
Females—*N* (%)	2405 (56.2%)	253 (60.8%)
Age—median (P25–P75)	39 (27–52)	37 (33–51)
Asthma—*N* (%)	1318 (30.8%)	148 (35.6%)
CSMS—median (P25–P75)	11.8 (5.8–21.1)	17.9 (9.1–32.2)
Medication classes used (%)		
INAH	94 (2.2%)	0 (0%)
INCS	1335 (31.2%)	99 (23.8%)
INAH+INCS	759 (17.8%)	45 (10.8%)
OAH	2733 (63.9%)	277 (66.6%)
Use of asthma medication—*N* (%)	977 (22.8%)	129 (31.0%)

Abbreviations: CSMS, Combined symptom‐medication score; INAH, Intranasal antihistamines; INCS, Intranasal corticosteroids; OAH, Oral antihistamines; P25–P75, Percentiles 25–75; SD, Standard‐deviation.

OAH were the most frequently reported medication class (*n* = 2733 users with EQ‐5D VAS data; *n* = 277 users with utility data, 63.9%), followed by INCS (*n* = 1335 with VAS data; *n* = 99 with utility data, 66.6%) and INAH+INCS (*n* = 759 with VAS data; *n* = 45 with utility data, 17.8%) (Table 1). The INCS whose use was most frequently reported (i.e., days of medication use) were mometasone (*n* = 14,451 VAS data; *n* = 104 utility observations). Among OAH, bilastine was the most common (*n* = 8341 VAS data; *n* = 81 utility observations), followed by desloratadine (*n* = 7545 VAS data; *n* = 93 utility observations) and rupatadine (*n* = 5156 VAS data; *n* = 205 utility observations) (Tables 3 and 4).

### 3.2 Class‐Level Comparisons

The comparative effects of different medication classes on health‐related quality of life are shown in Table 2. The use of INAH + INCS was associated with the most favourable outcomes, yielding higher EQ‐5D VAS scores than OAH (mean difference = 1.939, 95% CI: 1.355–2.523). In contrast, INAH monotherapy was associated with the least favourable results, showing lower VAS scores compared to all other classes: INAH+INCS (mean difference = −7.413, 95% CI: −9.723 to −5.097), INCS (mean difference = −6.571, 95% CI: −9.137 to −4.005), and OAH (mean difference = 0.565, 95% CI: −1.869 to 2.999). For the utility scores, INCS tended to be associated with higher utilities than INAH, while INAH+INCS tended to be associated with higher utilities than INAH, OAH and INCS.

**TABLE 2:** Results of multivariable regression models comparing different treatment classes on (A) EQ‐5D VAS (scale between 0 and 100) and (B) Utility (scale anchored at 0 and 1)^a^.

Comparison	Mean difference in EQ‐5D VAS levels (95% CI) [*N* days]	Mean difference in EQ‐5D utility levels (95% CI) [*N* days]
INAH vs. INCS	−3.750 (−5.839; −1.661) [400 vs. 22,377]	NA
INAH vs. INAH + INCS	−8.731 (−11.218; −6.244) [400 vs. 11,037]	NA
INAH vs. OAH	1.890 (0.438; 4.218) [400 vs. 36,159]	NA
INCS vs. INAH + INCS	−0.100 (−0.800; 0.600) [22,377 vs. 11,037]	−0.027 (−0.064; 0.010) [186 vs. 61]
INCS vs. OAH	0.415 (−0.050; 0.880) [22,377 vs. 36,159]	0.008 (−0.016; 0.032) [186 vs. 595]
INAH + INCS vs. OAH	1.900 (1.316; 2.484) [11,037 vs. 36,159]	0.022 (−0.015; 0.059) [61 vs. 595]

Abbreviations: INAH, Intranasal antihistamines; INAH + INCS, Fixed combination of intranasal antihistamines and corticosteroids; INCS, Intranasal corticosteroids; OAH, Oral antihistamines; VAS, Visual analogue scale.

^a^ A negative value indicates that the first intervention in the comparison is associated with a lower quality of life (VAS EQ‐5D or utility) than the second one. A positive value indicates that the first intervention in the comparison is associated with a higher quality of life (VAS or utilities) than the second one.

### 3.3 Individual Intranasal Medications

Analysis of individual intranasal medications (Table 3) revealed that mometasone was associated with a lower EQ‐5D VAS score compared to budesonide (mean difference = −5.288, 95% CI: −7.498 to −3.079). For the utilities, only the comparison between azelastine‐fluticasone and mometasone had more than 50 observations per intervention, and that comparison favoured azelastine‐fluticasone.

**TABLE 3:** Results of multivariable regression models comparing intranasal medications on EQ‐5D VAS (scale between 0 and 100).

Comparison	Mean difference in EQ‐5D VAS levels (95% CI) [*N* days]^a^
Azelastine‐fluticasone vs. Budesonide	−0.531 (−2.732; 1.670) [10,371 vs. 1307]
Azelastine‐fluticasone vs. Fluticasone furoate	−2.954 (−4.365; −1.542) [10,371 vs. 4025]
Azelastine‐fluticasone vs. Fluticasone propionate	−1.186 (−3.346; 0.974) [10,371 vs. 1695]
Azelastine‐fluticasone vs. Mometasone	0.642 (−0.196; 1.479) [10,371 vs. 14,451]
Azelastine‐fluticasone vs. Olopatadine‐mometasone	0.741 (−2.397; 3.878) [10,371 vs. 666]
Budesonide vs. Fluticasone furoate	2.478 (−1.369; 6.326) [1307 vs. 4025]
Budesonide vs. Fluticasone propionate	3.635 (−1.068; 8.337) [1307 vs. 1695]
Budesonide vs. Mometasone	5.288 (3.079; 7.498) [1307 vs. 14,451]
Budesonide vs. Olopatadine‐mometasone	−0.268 (−4.071; 3.535) [1307 vs. 666]
Fluticasone furoate vs. Fluticasone propionate	2.556 (−1.033; 6.145) [4025 vs. 1695]
Fluticasone furoate vs. Mometasone	1.423 (−0.105; 2.952) [4025 vs. 14,451]
Fluticasone furoate vs. Olopatadine‐mometasone	−2.486 (−7.665; 2.694) [4025 vs. 666]
Fluticasone propionate vs. Mometasone	0.707 (−2.726; 4.140) [1695 vs. 14,451]
Fluticasone propionate vs. Olopatadine‐mometasone	−3.479 (−10.039; 3.081) [1695 vs. 666]
Mometasone vs. Olopatadine‐mometasone	−0.960 (−4.333; 2.412) [14,451 vs. 666]

Abbreviation: VAS, Visual analogue scale.

^a^ A negative value indicates that the first intervention in the comparison is associated with a lower quality of life (VAS EQ‐5D) than the second one. A positive value indicates that the first intervention in the comparison is associated with a higher quality of life (VAS) than the second one.

### 3.4 Individual Oral Medications

For OAH, the results for EQ‐5D VAS showed that cetirizine was associated with a higher value than fexofenadine (mean difference: 5.116, 95% CI: 2.824–7.390) (Table 4). Furthermore, levocetirizine demonstrated a higher VAS score when compared to fexofenadine (4.741, 95% CI: 2.060–7.422) and loratadine (3.840, 95% CI: 1.930–5.749). On the other hand, rupatadine was also associated with a higher VAS score than levocetirizine (3.168, 95% CI: 0.808–5.528).

**TABLE 4:** Results of multivariable regression models comparing oral antihistamines on (A) EQ‐5D VAS (scale of 0 to 100) and (B) Utility (scale anchored between 0 and 1)^a^.

Comparison	Mean difference in EQ‐5D VAS levels (95% CI) [*N* days]	Mean difference in EQ‐5D utility levels (95% CI) [*N* days]
Bilastine vs. Cetirizine	1.348 (−0.408; 3.103) [8341 vs. 5188]	−0.026 (−0.077; 0.025) [81 vs. 72]
Bilastine vs. Desloratadine	1.431 (−0.033; 2.896) [8341 vs. 7545]	−0.037 (−0.082; 0.008) [81 vs. 93]^b^
Bilastine vs. Ebastine	0.472 (−1.532; 2.476) [8341 vs. 5426]	NA
Bilastine vs. Fexofenadine	0.555 (−1.841; 2.952) [8341 vs. 1581]	NA
Bilastine vs. Levocetirizine	−1.378 (−3.426; 0.670) [8341 vs. 2700]	NA
Bilastine vs. Loratadine	2.302 (−0.193; 4.797) [8341 vs. 1892]	NA
Bilastine vs. Rupatadine	−0.466 (−1.817; 0.885) [8341 vs. 5156]	0.020 (−0.050; 0.090) [81 vs. 205]
Cetirizine vs. Desloratadine	−1.479 (−3.212; 0.253) [5188 vs. 7545]	−0.009 (−0.050; 0.032) [72 vs. 93]^b^
Cetirizine vs. Ebastine	−1.798 (−4.215; 0.619) [5188 vs. 5426]	NA
Cetirizine vs. Fexofenadine	5.116 (2.842; 7.390) [5188 vs. 1581]	NA
Cetirizine vs. Levocetirizine	2.940 (1.036; 4.845) [5188 vs. 2700]	NA
Cetirizine vs. Loratadine	0.363 (−1.368; 2.094) [5188 vs. 1892]	NA
Cetirizine vs. Rupatadine	0.889 (−1.283; 3.061) [5188 vs. 5156]	0.036 (−0.027; 0.099) [72 vs. 205]
Desloratadine vs. Ebastine	1.104 (−0.964; 3.172) [7545 vs. 5426]	NA
Desloratadine vs. Fexofenadine	−1.935 (−4.240; 0.370) [7545 vs. 1581]	NA
Desloratadine vs. Levocetirizine	−1.281 (−2.675; 0.112) [7545 vs. 2700]	NA
Desloratadine vs. Loratadine	0.154 (−1.501; 1.809) [7545 vs. 1892]	NA
Desloratadine vs. Rupatadine	0.785 (−1.397; 2.968) [7545 vs. 5156]	0.054 (−0.005; 0.112) [93 vs. 205]^b^
Ebastine vs. Fexofenadine	−0.283 (−3.628; 3.061) [5426 vs. 1581]	NA
Ebastine vs. Levocetirizine	−1.310 (−3.784; 1.164) [5426 vs. 2700]	NA
Ebastine vs. Loratadine	−0.508 (−3.456; 2.439) [5426 vs. 1892]	NA
Ebastine vs. Rupatadine	−0.040 (−2.348; 2.268) [5426 vs. 5156]	NA
Fexofenadine vs. Levocetirizine	−4.741 (−7.422; −2.060) [1581 vs. 2700]	NA
Fexofenadine vs. Loratadine	1.117 (−2.329; 4.563) [1581 vs. 1892]	NA
Fexofenadine vs. Rupatadine	0.310 (−3.138; 3.758) [1581 vs. 5156]	NA
Levocetirizine vs. Loratadine	3.840 (1.930; 5.749) [2700 vs. 1892]	NA
Levocetirizine vs. Rupatadine	−3.168 (−5.528; −0.808) [2700 vs. 5156]	NA
Loratadine vs. Rupatadine	1.228 (−1.132; 3.587) [1892 vs. 5156]	NA

Abbreviation: VAS, Visual analogue scale.

^a^ A negative value indicates that the first intervention in the comparison is associated with a lower quality of life (VAS EQ‐5D or utility) than the second one. A positive value indicates that the first intervention in the comparison is associated with a higher quality of life (VAS or utilities) than the second one.

^b^ Full model did not converge. Results for the models without adjusting for day‐of‐week registration patterns.

## 4 Discussion

This study evaluated patients with self‐reported AR and compared the impact of different medication classes and individual medications on generic quality of life using both utility scores and VAS EQ‐5D. Overall, our results consistently show that fixed combinations of INAH + INCS are associated with a higher quality of life than INAH and OAH alone. INAH monotherapy was associated with lower quality‐of‐life scores than all other medication classes. At the individual drug level, mometasone was associated with a lower EQ‐5D VAS than budesonide and fluticasone furoate, while fexofenadine and levocetirizine tended to be associated with lower VAS values than other OAH.

The combined analysis of utilities and EQ‐5D VAS highlights their complementary roles, as shown in previous works [27, 28, 29]. Utilities derived from population preferences are essential for cost‐utility analyses and health technology assessments [30, 31]. The use of the EQ‐5D‐5L to derive these utilities is supported by recent, high‐quality evidence. A systematic review found high certainty of sufficient construct validity for the EQ‐5D in patients with allergic rhinitis [32]. While this confirms its validity, some real‐world studies, including MASK‐air analyses, have shown that the five‐level questions of EQ‐5D‐5L may underrepresent the burden of AR compared with disease‐specific tools such as the Rhinoconjunctivitis Quality of Life Questionnaire (RQLQ) [29, 33]. In contrast, the EQ‐5D VAS is often more sensitive to short‐term, daily variations in perceived health and may better capture the impact of AR symptoms [19, 29]. In this context, even though utility values are usually preferred and recognised as more rigorous, some authors have accepted the use of VAS values in cost‐utility analysis [34, 35].

Most randomised controlled trials in AR have traditionally focused on symptom reduction and improvements in disease‐specific quality of life (e.g., RQLQ) rather than on preference‐based measures such as utility scores [8, 9, 10]. Consequently, comparative effectiveness data for AR treatments in terms of utility gains remain scarce. Beyond the academic literature, there is also limited information from industry‐sponsored pharmacoeconomic studies addressing the cost‐effectiveness of AR interventions. For example, a health technology assessment by the Canadian Agency for Drugs and Technologies in Health suggested that azelastine–fluticasone was associated with higher incremental utility compared with monotherapies such as INAH and INCS [36]. However, while this assessment did not disclose quantitative utility data, estimates computed by our team based on the costs and cost‐effectiveness data provided are consistent with those reported in the present study. Specifically, this pharmacoeconomic report on azelastine‐fluticasone estimated positive incremental Quality‐Adjusted Life Years (QALY) gains relative to its components over a 14‐day cycle. We estimated that, in the Canadian report, the azelastine‐fluticasone combination demonstrated a QALY difference of 0.0004 compared to Fluticasone Propionate and 0.0005 compared to Azelastine. In our study, we observed differences of higher magnitude (i.e., a larger effect), consistent with INAH+INCS being associated with a higher quality of life than INCS (higher utilities) and INAH (we were only able to quantify differences in EQ‐5D VAS due to insufficient data on days with use of INAH and with utility information). Thus, our findings provide novel, real‐world utility estimates that complement and expand upon this limited prior evidence base, helping fill this evidence gap.

At the individual medication level, differences were mostly small, and this might be related to patients' satisfaction with treatment. In our studies, patients mostly use as‐needed treatment and increase their treatment when they are not satisfied. Thus, satisfaction is higher on monotherapy days than on co‐medication days, suggesting that patients try different treatments when not feeling sufficiently well controlled [37]. In line with previous work, we also observed that monotherapy days were associated with higher median VAS satisfaction levels, VAS EQ‐5D levels, and utilities (consistent with the median utility levels observed in patients with good control of allergic rhinitis) (Table S1) [27, 38]. Utility values are also in line with other recent cross‐sectional studies, such as a large German study that reported an average EQ‐5D‐5L index of 0.85 in patients with allergic respiratory diseases [39].

This study has some limitations. The most important was the insufficient sample size for most comparisons of individual medications' utilities. This precluded several analyses, led to lower precision in the estimates, and may reflect study bias with an over‐representation of those most frequently using the MASK‐air app (and which may be systematically different from the remaining patients). Country‐level representation was uneven, reflecting app usage patterns, regional differences in over‐the‐counter availability, or physician recommendations for specific medications. Our analysis was designed to mitigate these issues by focusing on individual‐level effects, adjusting for key confounders, and treating participants as random effects.

Reliance on self‐reported data introduces the potential for misclassification of covariates. We were unable to obtain information on AR control levels immediately prior to medication use, which is a potentially key determinant of treatment patterns [37, 40]. Indeed, previous studies suggest that patients often use medication intermittently, typically when they perceive their symptoms to be less well controlled [37]. To mitigate this issue, we used the CSMS levels of the day before as a proxy variable for AR control shortly before drug use. While the previous day's CSMS is supposed to be connected to pre‐medication symptoms, it is important to stress that the intensity of AR symptoms can fluctuate daily (even within the same individual) depending on various factors, including exposure to pollen or pollutants. Additionally, although the EQ‐5D VAS is validated, it is a generic measure and may be less sensitive to AR‐specific changes than disease‐specific PROMs. Despite adjustment for key confounders, residual confounding from unmeasured factors—such as socioeconomic status, treatment adherence, or comorbidities beyond asthma—cannot be excluded.

This study also has some strengths, including the use of patient‐reported data directly entered into an mHealth platform, which facilitates prospective, standardised and high‐volume patient reporting across diverse geographical settings. The application of IPTW was a crucial step in mitigating confounding [41, 42]. Finally, to the best of our knowledge, this is the first study to compare AR medications on EQ‐5D VAS and utilities, allowing its results to be incorporated into cost‐utility studies. Cost‐utility studies are one type of full economic evaluation that allows simultaneous consideration of the costs and effectiveness of interventions, taking into account preferences for the underlying health states (as measured through utilities). These studies allow us to understand whether society is willing to pay the extra costs of a certain intervention to achieve the corresponding health gains. In this context, cost‐effectiveness is one of the criteria based on which interventions should be evaluated when formulating guideline recommendations in the GRADE Evidence‐to‐Decision framework [43, 44]. As a result, findings of this study are being used to inform the ARIA‐EAACI 2024–2025 guidelines on the cost‐effectiveness criterion.

In conclusion, this study compared different AR treatment classes and individual medications in terms of utilities and EQ‐5D VAS scores using patient‐reported data. It showed that (i) at the class level, INAH+INCS were consistently associated with better quality‐of‐life outcomes than OAH and INAH monotherapy, with concordant results across both utilities and EQ‐5D VAS scores; and (ii) at the individual drug level, differences were mostly small. By providing estimates of health utilities for AR treatments, the findings of this study can be used in cost‐utility analyses, thereby supporting evidence‐based decision‐making and contributing to informing ARIA‐EAACI guidelines.

## Author Contributions

Nuno Lourenço‐Silva, Bernardo Sousa‐Pinto and Rafael José Vieira participated in the study design, methodology, data analysis and manuscript writing. Jean Bousquet participated in the study design, methodology and manuscript review. All other authors participated in participant inclusion and manuscript review.

## Funding

This study did not receive specific funding. MASK‐air has been supported by EU grants (EU Structural and Development grant, POLLAR: EIT Health and Structural and Development Funds) and educational grants from Mylan‐Viatris, ALK, GSK, Novartis and Noucor.

## Conflicts of Interest

J.B. reports personal fees from Cipla, Menarini, Mylan, Novartis, Purina, Sanofi‐Aventis, Teva, Noucor, other from KYomed‐Innov, and other from Mask‐air‐SAS, outside the submitted work. L.K. reports personal fees from Allergopharma, personal fees from Viatris, personal fees from LETI Pharma, personal fees from Stallergenes, personal fees from ALK Abelló, personal fees from HAL Allergie, personal fees from Sanofi, personal fees from AstraZeneca, personal fees from GSK, personal fees from Inmunotek, personal fees from Regeneron Pharmaceuticals, personal fees from Novartis, outside the submitted work; and membership of AeDA, DGHNO, Deutsche Gesellschaft für Allergologie und klinische Immunologie, HNO‐BV, GPA, EAACI. T.Z. reports honoraria for lectures from Amgen, AstraZeneca, AbbVie, ALK‐Abelló, Almirall, Astellas, Bayer Health Care, Bencard, Berlin Chemie, FAES Farma, HAL Allergie GmbH, Henkel, Kryolan, Leti, L'Oreal, Meda, Menarini, Merck Sharp & Dohme, Novartis, Nuocor, Pfizer, Sanofi, Stallergenes, Takeda, Teva, UCB and Uriach; Fees for industry consulting were received from Abivax, Almirall, Bluprint, Celldex, Celltrion, Novartis and Sanofi; in addition he declares non‐paid organisational affiliations: Committee member, ‘Allergic Rhinitis and its Impact on Asthma’ (ARIA), Member of the Board, German Society for Allergy and Clinical Immunology (DGAKI), Head, European Centre for Allergy Research Foundation (ECARF), President, Global Allergy and Asthma Excellence Network (GA^2^LEN), and Member, Committee on Allergy Diagnosis and Molecular Allergology, World Allergy Organisation (WAO). N.G.P. reports grants from Nestle, Numil, Vianex, and Vibrant, and personal fees from Abbott, AstraZeneca, GSK, HAL, Menarini, Novartis, Danone Nutricia, OM Pharma, Regeneron, and Sanofi, outside the submitted work. J.S. reports grants and personal fees from Sanofi, personal fees from GSK, personal fees from Novartis, personal fees from AstraZeneca, personal fees from MUNDIPHARMA, personal fees from FAES FARMA, outside the submitted work. S.K.T.‐S. reports grants and other from GSK, grants and other from Sanofi, other from AstraZeneca, other from ALK‐Abelló, other from OrionPharma, outside the submitted work. D.E.L.‐L. reports personal fees from ALK, Armstrong, AstraZeneca national and global, Bayer, Chiesi, Grunenthal, Grin, GSK national and global, Viatris, Menarini, MSD, Novartis, Pfizer, Sanofi, Siegfried, Carnot, Syneos Health, grants from Abbvie, Bayer, Lilly, Sanofi, AstraZeneca, Pfizer, Novartis, Pulmonair, GSK, Chiesi, Biopharma, outside the submitted work; and Editor in chief of Immune System (Karger)Ðember of asthma committee ACAAIÐubgroup chair of allergen immunotherapy Practice parameter update JTF AAAAI/ACAAI 2024Ðember of allergen immunotherapy committee AAAAIÜhair of allergen immunotherapy committee CMICAÐember of allergic asthma task force EAACI. M.G. reports personal fees from Sanofi, and Thermo Fisher Scientific, outside the submitted work. O.P. reports grants and personal fees from ALK‐Abelló, grants and personal fees from Allergopharma, grants and personal fees from Stallergenes Greer, grants and personal fees from HAL Allergy Holding B.V./HAL Allergie GmbH, grants and personal fees from Bencard Allergie GmbH/Allergy Therapeutics, grants and personal fees from Laboratorios LETI/LETI Pharma, grants and personal fees from GlaxoSmithKline, personal fees from ROXALL Medizin, personal fees from Novartis, grants and personal fees from Sanofi‐Aventis and Sanofi‐Genzyme, personal fees from Med Update Europe GmbH, personal fees from streamedup! GmbH, personal fees from Pohl‐Boskamp, grants from Inmunotek S.L., personal fees from John Wiley and Sons, AS, personal fees from Paul‐Martini‐Stiftung (PMS), personal fees from Regeneron Pharmaceuticals Inc., personal fees from RG Aerztefortbildung, personal fees from Institut für Disease Management, personal fees from Springer GmbH, grants and personal fees from AstraZeneca, personal fees from IQVIA Commercial, personal fees from Ingress Health, personal fees from Wort&Bild Verlag, personal fees from Verlag ME, personal fees from Procter&Gamble, personal fees from ALTAMIRA, personal fees from Meinhardt Congress GmbH, personal fees from Deutsche Forschungsgemeinschaft, personal fees from Thieme, grants from Deutsche AllergieLiga e.V., personal fees from AeDA, personal fees from Alfried‐Krupp Krankenhaus, personal fees from Red Maple Trials Inc., personal fees from Königlich Dänisches Generalkonsulat, personal fees from Medizinische Hochschule Hannover, personal fees from ECM Expro&Conference Management, personal fees from Technical University Dresden, grants and personal fees from Lilly, personal fees from Japanese Society of Allergy, personal fees from Forum für Medizinische Fortbildung, personal fees from Dustri‐Verlag, personal fees from Pneumolive, grants and personal fees from ASIT Biotech, personal fees from LOFARMA, personal fees from Paul‐Ehrlich‐Institut, personal fees from Almirall, personal fees from BREAZY Health, personal fees from Biologix, outside the submitted work; and Vice President and member of EAACI Excom, member of ext. board of directors DGAKI; coordinator, main‐ or co‐author of different position papers and guidelines in rhinology, allergology and allergen immunotherapy; Editor‐in‐Chief (EIC) of Clinical Translational Allergy(CTA), Associate Editor (AE) of Allergy. L.T.‐B. reports personal fees from Sanofi and LETI, outside the submitted work. The other authors have nothing to declare, outside the submitted paper.

## Supporting information


**Figure S1:** Flow diagram illustrating participants' selection.
**Table S1:** Comparison of the patient‐reported outcome measures in monotherapy versus in co‐medication.
**Table S2:** Visual analogue scales (VASs) used for daily monitoring the impact of allergic rhinitis and asthma symptoms in MASK‐air.
**Table S3:** Frequency of MASK‐air users and daily monitoring questionnaire days per country for the EQ‐5D VAS and Utility samples.

## Data Availability

The data that support the findings of this study are available from the corresponding author upon reasonable request.
