# Supplementary material for: Comparison of Allergic Rhinitis Treatments on Utilities and Quality of Life: A MASK‐air Study
Source: Clin Exp Allergy. 2026 May 6;56(8):904–13. doi: 10.1111/cea.70335 (PMC13429351; doi:10.1111/cea.70335)
Supplement: Supplementary file 1 — Figure S1: Flow diagram illustrating participants' selection. Table S1: Comparison of the patient‐reported outcome measures in monotherapy versus in co‐medication. Table S2: Visual analogue scales (VASs) used for daily monitoring the impact of allergic rhinitis and asthma symptoms in MASK‐air. Table S3: Frequency of MASK‐air users and daily monitoring questionnaire days per country for the EQ‐5D VAS and Utility samples. [file CEA-56-904-s001.docx]

**Supplementary Material**

**Supplementary Figures**

**Supplementary Figure 1. Flow diagram illustrating participants selection**

Days of rhinitis medication (monotherapy) of MASK-air users aged 16*-75 years with valid EQ-5D-5L data

[May 15th, 2015 – December 31st, 2024]

(i) EQ-5D VAS – *N* users = 7885; *N* days = 106,852)

(ii) Utilities – *N* users =1262; *N* days = 1949)

Days of rhinitis medication of MASK-air users aged 16*- 75 years [May 15th, 2015 – December 31st, 2024] and for which there was availability of (i) EQ-5D VAS or (ii) Utilities, and (iii) information of rhinitis control on the previous day

(i) EQ-5D VAS – *N* users = 4276; *N* days = 69,973

(ii) Utilities – *N* users = 416; *N* days = 842

*or lower (not below 13 years old) for countries where the digital age of consent is lower

**Excluded for not having available information on rhinitis control on the previous day**

(i) EQ-5D VAS *– N* users = 3609; *N* days = 36,879

(ii) Utilities *– N* users = 846; *N* days = 1107

**Supplementary Tables**

**Supplementary Table 1. Comparison of the patient reported outcome measures in monotherapy *versus* in co-medication**

|  | **Monotherapy** | | | **Co-medication** | |
| --- | --- | --- | --- | --- | --- |
|  | **OAH** | **INCS** | **INAH+INCS** | **INCS+OAH** | **INAH+INCS+OAH** |
| **N days** | 56,699 | 33,080 | 17,489 | 33,847 | 19,328 |
| **VAS nose (maximum) – median (P25-P75)** | 56 (26-84) | 45 (21-71) | 44 (20-68) | 60 (32-84) | 60 (32-84) |
| **VAS nose (median) – median (P25-P75)** | 33 (13-64) | 25 (10-51) | 22 (8-46) | 37.2 (15-64) | 32 (14-61) |
| **CSMS (maximum) – median (P25-P75)** | 34.3 (18.8-50.1) | 28.3 (16.3-44.0) | 28.0 (15.6-42.1) | 41.3 (25.1-57.2) | 40.1 (24.7-56.3) |
| **CSMS (median) – median (P25-P75)** | 22.6 (10.0-39.8) | 18.4 (10.0-33.3) | 17.5 (9.4-29.9) | 28.6 (15.3-45.7) | 26.3 (14.3-42.1) |
| **VAS satisfaction – median (P25-P75)** | 81 (57-92) | 86 (66-93) | 81 (63-91) | 81 (60-92) | 73 (36-90) |
| **VAS EQ-5D – median (P25-P75)** | 82 (68-92) | 85 (71-93) | 85 (70-94) | 81 (64-92) | 78 (55-90) |
| **Utilities – median (P25-P75)** | 0.91 (0.82-0.98) | 0.92 (0.85-1.00) | 0.94 (0.88-1.00) | 0.91 (0.80-0.96) | 0.78 (0.72-0.91) |

CSMS=Combined symptom-medication score; INAH=Intranasal antihistamines; INCS=Intranasal corticosteroids; OAH=Oral antihistamines; P25-P75: Percentiles 25-75; VAS=Visual Analogue Scale; SD=Standard-deviation

**Supplementary Table 2. Visual analogue scales (VASs) used for daily monitoring the impact of allergic rhinitis and asthma symptoms in MASK-air^®^**

| **VAS** | **Question** |
| --- | --- |
| VAS Global Allergy Symptoms | Overall, how much are your allergic symptoms bothering you today? |
| VAS Nose | How much are your nose symptoms bothering you today? |
| VAS Eyes | How much are your eye symptoms bothering you today? |
| VAS Asthma | How much are your asthma symptoms bothering you today? |

**Supplementary Table 3. Frequency of MASK-air^®^ users and daily monitoring questionnaire days per country for the EQ-5D VAS and Utility samples.**

| **Country** | ***N* days (N users)** | |
| --- | --- | --- |
|  | **EQ-5D VAS** | **Utility** |
| Argentina | 1491 (144) | Not included |
| Australia | 43 (15) | 4 (4) |
| Austria | 463 (40) | 1 (1) |
| Belgium | 384 (16) | 37 (3) |
| Brazil | 928 (106) | Not included |
| Canada | 19 (6) | 1 (1) |
| Czech Republic | 2391 (139) | 2 (2) |
| Denmark | 16 (4) | 1 (1) |
| Ecuador | 158 (8) | Not included |
| Finland | 809 (20) | 10 (7) |
| France | 6761 (595) | 75 (56) |
| Germany | 13,483 (587) | 163 (81) |
| Great Britain | 509 (38) | 27 (16) |
| Greece | 3432 (267) | 12 (6) |
| Hungary | 3097 (191) | Not included |
| Italy | 5332 (383) | 85 (55) |
| Japan | 219 (33) | Not included |
| Lebanon | 165 (21) | Not included |
| Lithuania | 6784 (334) | 196 (20) |
| Mexico | 1771 (133) | 40 (21) |
| Netherlands | 399 (29) | 30 (24) |
| Poland | 6526 (391) | 54 (44) |
| Portugal | 7931 (382) | 62 (47) |
| Slovenia | 1469 (91) | 1 (1) |
| Spain | 3272 (189) | 37 (24) |
| Sweden | 72 (7) | 2 (2) |
| Switzerland | 577 (38) | 5 (3) |
| Turkey | 1383 (61) | Not included |
| Ukraine | 75 (5) | Not included |
| United States | 14 (3) | Not included |
